# Assessing the Performance of Sensor Fusion Methods: Application to Magnetic-Inertial-Based Human Body Tracking

**DOI:** 10.3390/s16020153

**Published:** 2016-01-26

**Authors:** Gabriele Ligorio, Elena Bergamini, Ilaria Pasciuto, Giuseppe Vannozzi, Aurelio Cappozzo, Angelo Maria Sabatini

**Affiliations:** 1 The BioRobotics Institute, Scuola Superiore Sant’Anna, Piazza Martiri della Libertà 33, 56125 Pisa, Italy; angelo.sabatini@sssup.it; 2 Department of Movement, Human and Health Sciences, Interuniversity Centre of Bioengineering of the Human Neuromusculoskeletal System, University of Rome “Foro Italico”, Piazza Lauro de Bosis 15, 00135 Roma, Italy; elena.bergamini@uniroma4.it (E.B.); ilaria.pasciuto@uniroma4.it (I.P.); giuseppe.vannozzi@uniroma4.it (G.V.); aurelio.cappozzo@uniroma4.it (A.C.)

**Keywords:** sensor fusion, algorithm benchmarking, inertial-magnetic sensors, human motion tracking, orientation, locomotion

## Abstract

Information from complementary and redundant sensors are often combined within sensor fusion algorithms to obtain a single accurate observation of the system at hand. However, measurements from each sensor are characterized by uncertainties. When multiple data are fused, it is often unclear how all these uncertainties interact and influence the overall performance of the sensor fusion algorithm. To address this issue, a benchmarking procedure is presented, where simulated and real data are combined in different scenarios in order to quantify how each sensor’s uncertainties influence the accuracy of the final result. The proposed procedure was applied to the estimation of the pelvis orientation using a waist-worn magnetic-inertial measurement unit. Ground-truth data were obtained from a stereophotogrammetric system and used to obtain simulated data. Two Kalman-based sensor fusion algorithms were submitted to the proposed benchmarking procedure. For the considered application, gyroscope uncertainties proved to be the main error source in orientation estimation accuracy for both tested algorithms. Moreover, although different performances were obtained using simulated data, these differences became negligible when real data were considered. The outcome of this evaluation may be useful both to improve the design of new sensor fusion methods and to drive the algorithm tuning process.

## 1. Introduction

Sensor fusion is a signal processing technique that combines data measured by multiple sources in order to create a single measurement system with an augmented performance over each standalone sensor [1,2]. The reason for designing sensor fusion algorithms (SFAs) is two-fold: first, to improve the accuracy and/or robustness of the outcome by exploiting data redundancy and/or complementarity; second, to provide a complete picture of the phenomenon under investigation unifying the partial observations provided by each sensor.

SFAs are widely employed in several applications including autonomous navigation, robotics, environmental monitoring, and healthcare [2,3,4,5]. In particular, the sensor fusion based on observations from magnetic and inertial sensors (commonly referred to as Magnetic-Inertial Measurement Units, MIMUs) is increasingly employed for the estimation of human body segment orientation in movement analysis and related applications [6,7,8]. A MIMU embeds a tri-axial gyroscope, accelerometer, and magnetic sensor that provide measurements of the body’s angular velocity, the specific force (*i.e.*, the sum of the external and gravitational accelerations), and the Earth’s magnetic field, respectively. The 3D body orientation can be estimated through the numerical integration of the rotational kinematic equation of a rigid body, by using angular velocity measurements and known initial conditions. The accelerometer and the magnetic sensors are supposed to track two external reference vectors: the Earth gravity acceleration (vertical reference vector) and the Earth magnetic field (heading reference vector). Estimating the body orientation by comparing the reference vectors measured in the body frame with their known counterparts expressed in an inertial reference frame, is known as field vector matching [9]. Usually, gyroscope integration and field vector matching are combined in an SFA to purposefully exploit their complementary properties. Several SFAs have been successfully proposed in the literature for the MIMU-based orientation estimation, which typically rely on either Kalman filtering [9,10,11,12] or complementary filtering [13,14]. On the contrary, when used alone, these sensors may yield poor results due to different issues characterizing the magnetic and the inertial sensors.

In this respect MIMU observations are disparate [3], in the sense that part of the orientation information is observed in three different physical domains, *i.e.*, the angular velocity, the specific force, and the Earth magnetic field vector. For this reason, MIMU data may be considered uncorrelated to each other, *i.e.*, no crosstalk among the three domains is expected. The only exception is the gyroscope sensitivity to the gravity and external accelerations [15] which, however, is often considered negligible as compared to other gyroscope error sources [16]. Within each domain, measurement noise is present in the sensor outputs [3], *i.e.*, each sample of measured angular velocity, specific force, and magnetic field exhibits a degree of uncertainty [17] which has different effects on the orientation estimates due to the different ways the MIMU sensor measurements are used to this purpose. Gyroscope-based tracking proved to be accurate during short-term rapid movements, although it is prone to boundless drift error over time occurring during integration. Of great concern in this regard is also the gyroscope bias, a slowly-varying output that is present even when the gyroscope is still. On the other hand, field vector matching does not suffer from drift errors, but it is heavily affected by external accelerations and magnetic disturbances, which are continuous and time-varying. Therefore, measurement noise, sensor bias, and external factors (external accelerations and ferromagnetic disturbances) result in conflicting information to be fused in the SFA, which may lead to highly inaccurate estimates of the 3D body orientation and even to the SFA failure to converge [3].

Unfortunately, when sensor observations are fused in an SFA, it is very difficult to assess to which extent each sensor issue influences the final error. This information would be crucial to guide the SFA design process (*i.e.*, the choice of different tuning settings or of the adaptive mechanisms to be built in the SFA) or to compare different combinations of sensor hardware components. Therefore, the development of benchmarking methods aimed at quantifying the effect of each sensor issue on the SFA performance is of the utmost importance. Nevertheless, no clear methodologies, guidelines, or tools for SFA performance assessment are available.

The main contribution of this paper is to propose a novel benchmarking method for the assessment of SFAs’ performance. Another subsidiary contribution is to provide, as an outcome of the proposed benchmarking method, useful considerations about the MIMU-based orientation estimation research field. In fact, to show the potentialities of the proposed method and to illustrate its application, a case study is considered where the 3D orientation of a human body segment is estimated using a MIMU during a clinical test. The proposed benchmarking method relies on the concept of data hybridization: measured and simulated MIMU data are combined in different ways to create different testbeds, hereafter referred to as scenarios. Each scenario is conceived to isolate the influence of the errors characterizing each sensor and to allow quantifying the efficacy of adaptive mechanisms built in different SFAs. In order to highlight both these specific aspects of the benchmarking problem, two different Kalman filter-based SFAs were considered and their performance in estimating the 3D orientation of the pelvis during a Timed Up and Go test was assessed.

## 2. Methods

### 2.1. Proposed Benchmarking Method: Overview

**Figure 1:**
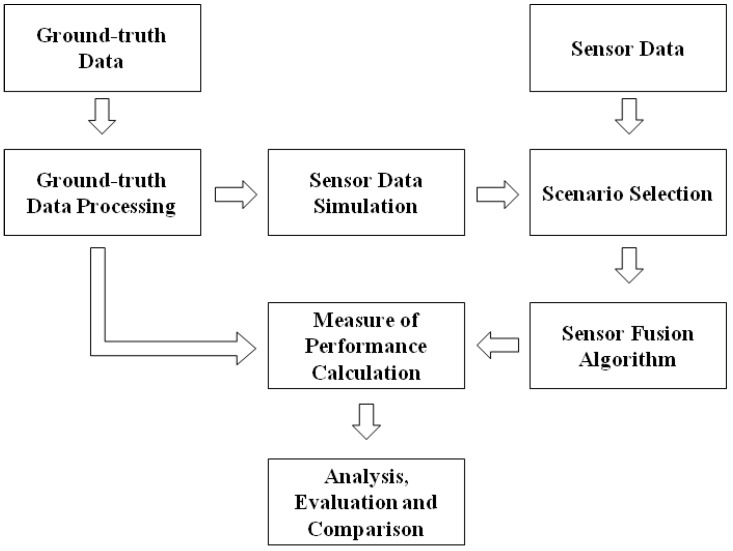
General overview of the proposed SFA evaluation framework.

The proposed method for evaluating and comparing the performance of SFAs is based on the workflow depicted in Figure 1.

Specifically, for the application investigated in the present study, ground-truth and real sensor data were acquired during a motion capture session. The ground-truth data were processed within the Ground-truth Data Processing block to obtain the reference output, which was used to simulate ideal sensor data (Data Simulation block). In addition it provided a reference for the calculation of the Measure Of Performance (MOP), *i.e.*, the metrics that quantifies the performance of an algorithm. A pool of evaluation scenarios (Scenario Selection block) was then created. Each scenario is a different combination of measured and/or simulated MIMU data. The SFA under analysis was then run using the data of each scenario as input, and the MOP was calculated by comparing the corresponding output with the reference one.

### 2.2. The Timed up and Go Dataset

**Figure 2:**
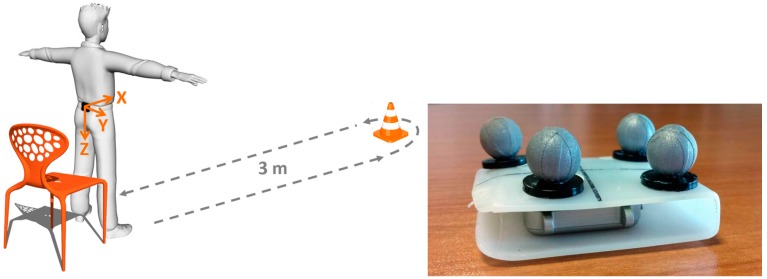
(**Left**) Sensor location on the participants’ body, axes orientation and TUG scheme, and (**Right**) Opal MIMU with the infrared reflective marker cluster.

The considered case study is the estimation of the pelvis orientation, by using data measured by a waist-worn MIMU, during a Timed Up and Go test (TUG) (Figure 2), which is a frequently performed clinical test aimed at assessing motor function. In particular, the TUG test was selected because it is a complex motor task, being characterized by transitory phases, such as sit-to-stand or change of direction, and by cyclic movements, such as level walking.

The University ethics board approved the adopted experimental protocol: 24 healthy subjects, after being informed of the goals and the modalities of the experiments, performed a TUG test. Prior to the TUG test, a MIMU (Opal, APDM Inc., Portland, OR, USA), embedding a tri-axial gyroscope, accelerometer, and magnetic sensor (±6 g with g = 9.81 m/s^2^, ±1500°/s and ±600 μT of full-range scale, respectively), was secured to the participants’ lower back (lumbar region of the spine, approximately at L3–L4 vertebrae level, Figure 2), using an elastic belt. MIMU data were collected at 128 samples/s. A plastic plaque equipped (Figure 2) with a cluster of four infrared reflective markers was rigidly attached to the MIMU case for ground-truth data acquisition using a nine-camera motion capture system (Vicon MX3, Oxford, UK) at 100 sample/s.

The reference frames used in this paper were the navigation frame {n} (also referred to as the global or inertial frame) and the body frame {b} (also referred to as the local frame). In the following, $p^{b}$ denotes the representation of the generic vector p in {b}, whereas $q^{bn}$ and $R^{bn}$ represent, respectively, the quaternion of orientation and the orientation matrix which rotate {n} to {b}. Furthermore, $\hat{p}$ is an estimate of the vector p.

The MIMU sensor was calibrated before the experiments, as suggested in [6]. The navigation reference frame was defined with the *z*-axis aligned with the vertical direction, whereas the angle about the vertical axis was set as the value recorded during a static posture performed at the beginning of the trial. This static time window, which lasted 3 s, was also used to perform the gyroscope bias capture [6]. MIMU and stereophotogrammetric data streams were electronically synchronized using a square wave signal simultaneously detected by both systems. Both data were resampled at 200 Hz using cubic spline interpolation. Marker trajectories were low-pass filtered using a second-order zero-lag Butterworth filter. The cut-off frequency was determined by performing a residual analysis [18] on each trial of each subject and conservatively set to 6 Hz for all trials. A marker-cluster reference frame was then defined using the markers attached on the MIMU and its alignment with the MIMU case verified [19].

In order to obtain simulated (considered to be error-free and, thus, referred to as ideal) MIMU data, the ground-truth body orientation $q^{bn}$ and position $b^{n}$, obtained from the marker cluster attached to the MIMU case, were used as an input to the data simulator described in [20]. In particular, for each trial, the ideal angular velocity was obtained by differentiating the ground-truth quaternions $q^{bn}$ with standard formulae [21]. The same quaternions were used to rotate the coordinates of the Earth’s gravitational and magnetic field vectors, known *a priori*, from {n} to {n}. The rotated Earth’s magnetic field represented the ideal magnetometer data, whereas the external acceleration was added to the rotated gravitational field to build the ideal accelerometer data. All the ideal sensor data were corrupted with white noise before applying the stochastic filters (standard deviation estimated during the static postures equal to 5 × 10^−3^ rad/s, 5 × 10^−3^ m/s^2^ and 0.15 µT). At this point in time, both the experimental and the ideal data required to devise the different scenarios for the SFA evaluation were available.

### 2.3. Scenario Selection

To isolate the influence of the errors characterizing each MIMU sensor and to quantify the efficacy of the adaptive mechanisms built in to test SFAs, the following scenarios were defined (Table 1): (a) two scenarios where the full set of MIMU data was either simulated or measured (hereafter, they are referred to as the SIM and MEAS scenarios, respectively). These two scenarios represent the best and worst testbeds for the considered SFAs, respectively; (b) three scenarios (named GYR, ACC, and MAG) in which two MIMU sensors’ data were simulated and one MIMU sensor at a time was accounted for with its measured data. Specifically, the name of each scenario indicates the abbreviation of the sensor accounted for with its measured data. The rationale is to isolate the influence of each sensor data issue on the overall SFA output; (c) a scenario (named MOD) which differed from the SIM scenario in the way the simulated accelerometer data were generated: in this case, in fact, the simulated accelerometer data contained only the contribution due to the gravity reference vector (that determines the orientation estimation), without taking into account the external acceleration, which in this context represents a disturbance. In this way, the detrimental effect of the external acceleration on the SFAs performance was also evaluated.

**Table 1:** The six evaluation scenarios considered in this work, listed theoretically from the best to the worst case.

Scenario	Gyroscope	Accelerometer	Magnetometer
MOD	simulated	gravity-only	simulated
SIM	simulated	simulated	simulated
GYR	measured	simulated	simulated
ACC	simulated	measured	simulated
MAG	simulated	simulated	measured
MEAS	measured	measured	measured

### 2.4. Sensor Fusion Algorithms

**Figure 3:**
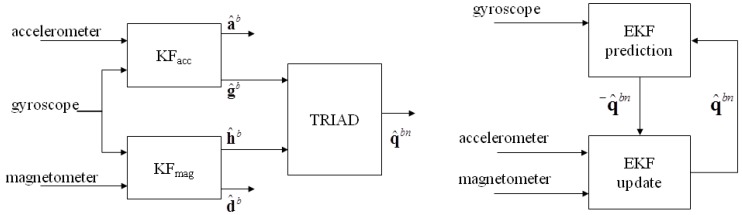
Overview of the Kalman-based SFAs considered in this work: (**Left**) Algorithm 1 and (**Right**) Algorithm 2.

Two Kalman-based orientation estimators were benchmarked in this work by using the proposed approach (Figure 3). The pseudo-code description of the two algorithms is reported in the Appendix A. In Algorithm 1, presented in [10], gyroscope measurements are used as input in two parallel linear Kalman filters that separate the component due to the reference field vectors (Earth’s gravitational $g^{b}$ and magnetic $h^{b}$ fields) from the disturbances affecting the accelerometer and magnetometer readings (the external acceleration $a^{b}$ and the magnetic disturbances $d^{b}$, respectively). The estimated reference field vectors ${\hat{g}}^{b}$ and ${\hat{h}}^{b}$ are then used to feed the TRIAD method [22] for single-frame orientation estimation, given that $g^{n}$ and $h^{n}$ are known.

Algorithm 2 is the Extended Kalman Filter (EKF) presented in [9]. In the prediction step, the quaternion estimate is projected ahead using the measured sample of the angular velocity. Then, assuming that external accelerations are negligible and no ferromagnetic disturbances are present, the measured acceleration and the measured magnetic field vector are used to update the projected quaternion in the nonlinear measurement equation. A linearization step is then needed at each iteration.

The combination of ideal and/or measured angular velocity, acceleration, and magnetic field vector data, associated with each above-mentioned scenario, were fed into both Algorithms 1 and 2. For each SFA and time sample, six orientation estimates were obtained (one for each scenario) and each of them was compared to the ground-truth body orientation, as explained in Section 2.5.

### 2.5. Measure of Performance

The accuracy in estimating the MIMU orientation was expressed in terms of orientation error computed as the quaternion that rotates the estimated MIMU orientation ${\hat{q}}_{k}^{bn}$ onto the ground-truth one $q_{k}^{bn}$ as proposed in [23]:

(1) $$\Delta {\hat{q}}_{k}^{bn}=q_{k}^{bn}⊗{\left({\hat{q}}_{k}^{bn}\right)}^{-1}$$

where ⊗ represents the quaternion multiplication operator and ${\left(q\right)}^{-1}$ is the inverse quaternion operator.

Then, in accordance with [10], $\Delta {\hat{q}}_{k}^{bn}$ was split into two error components, one related to the heading Δheadq^kbn (yaw angle) and one to the attitude Δattq^kbn (pitch and roll angles). The heading and attitude orientation parameterization, in fact, is particularly convenient for the problem at hand as the pitch and roll angles may be reasonably assumed to have similar error characteristics since they both represent an inclination with respect to the vertical direction [6,23]. The Root Mean Square (RMS) values of the scalar part (indicated as S in the following equations) of both Δheadq^kbn and Δattq^kbn were then considered as MOPs:

(2) RMShead=1N∑k=​1N(2acos[S(Δheadq^kbn)])2RMSatt=1N∑k=​1N(2acos[S(Δattq^kbn)])2

The MOP was computed for each scenario, SFA, and trial performed by each subject.

### 2.6. Statistical Analysis

In order to assess the performance of both Algorithms 1 and 2, the following specific questions were considered: (i) To which extent the SFA performance is affected by the errors characterizing each MIMU sensor? (ii) Can the SFA mitigate the effect of these sensor-specific errors, at least when all other sensors’ data are considered to be as ideal? (iii) How do different SFAs behave when fed with either simulated or real sensor signals?

For what concerns questions (i) and (ii) (intra-algorithm analysis), a one way repeated-measures ANalysis Of VAriance (ANOVA) was performed, using the scenario as a factor, on both the attitude and heading accuracy obtained for each method, separately. The MOP values were transformed according to the Tukey Ladder of Powers in order to achieve normality, in case of a lack of normality revealed by the Shapiro-Wilk test. The Greenhouse-Geisser correction was used to take into account possible violations of the sphericity assumption. Given possible significant differences revealed by the ANOVA test for the scenario effect, the Dunn-Sidak’s *post-hoc* pairwise tests were used to compare the GYR, ACC, and MAG scenarios with both the SIM and MEAS scenarios in order to answer (i) and (ii), respectively. In addition, the same test was adopted to compare the MOD and SIM scenario in order to assess the effect of the external acceleration on the SFAs output.

In order to answer question (iii) (inter-algorithm analysis), the presence of significant differences between the orientation accuracy obtained for Algorithms 1 and 2 was verified using a Wilcoxon test for both the SIM and MEAS scenarios. The alpha level of significance was set to 0.05 for all statistical tests.

## 3. Results

**Figure 4:**
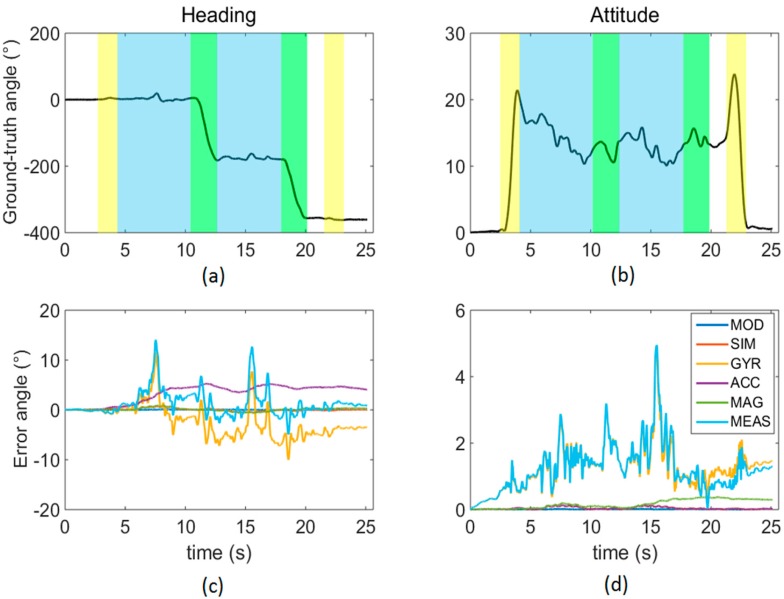
Heading (**a**) and attitude (**b**) ground-truth curves; heading (**c**) and attitude (**d**) error angles obtained for all the six scenarios considered. The colored bands in the upper row denote activities of sit-to-stand and stand-to-sit (**yellow**), walking (**blue**), and 180° turns around the cranio-caudal axis.

The heading and attitude ground-truth curves and the errors associated to each simulated scenario are reported in Figure 4 for one randomly-chosen participant. The different phases of the TUG task are also indicated by different colors: sit-to-stand and stand-to-sit (yellow bands), walking (light blue bands), and turns of 180° about the cranio-caudal direction (green bands).

**Figure 5:**
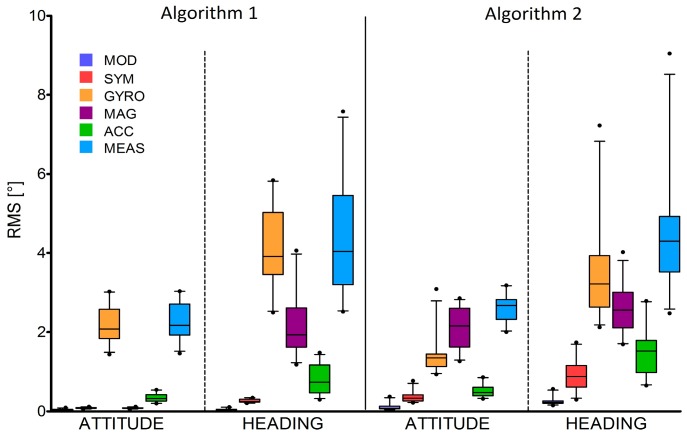
RMS_head_ and RMS_att_ median and inter quartile ranges obtained for the two considered SFAs and all the six tested scenarios.

Figure 5 shows the RMS error values (medians and inter-quartile ranges) obtained for both Algorithms 1 and 2.

For what concerns the intra-algorithm comparisons, the results of the one-way repeated measures ANOVA are reported in Table 2. A significant scenario effect was found for both heading and attitude errors and for both Algorithms 1 and 2. The scenario factor accounts for a minimum of 83% to a maximum of 97% of the overall orientation errors variance.

**Table 2:** Results of the one-way repeated measures ANOVA for both the attitude and heading errors and for each tested algorithm. Degrees of freedom for the effect (df_scenario_) and for the error term (df_error_) are reported together with *F* values, *p* values and partial eta squared (η^2^).

Algorithm	MOP	df_scenario_	df_error_	*F*	*p*	η^2^
1	RMS_att_	1.13	25.93	193.95	<0.001	0.85
	RMS_head_	3.09	71.21	661.30	<0.001	0.97
2	RMS_att_	1.05	24.26	113.60	<0.001	0.83
	RMS_head_	3.37	77.67	285.84	<0.001	0.92

The selection of the scenarios considered for the *post hoc* tests was done according to the questions presented in Section 2.6. In Table 3, the results of the statistical analysis are reported (mean differences between the errors obtained in the considered scenarios and significance).

**Table 3:** *Post*-*hoc* analysis: marginal differences between the scenarios indicated in the first and second column for both algorithms. Significant differences are indicated with an asterisk.

Tested Scenario	Testbed Scenario	Algorithm 1				Algorithm 2			
		Attitude		Heading		Attitude		Heading	
GYR	SIM	2.11	*	3.82	*	1.03	*	2.61	*
	MEAS	−0.9	*	−0.19		−1.22	*	−0.98	*
ACC	SIM	0.25	*	0.54	*	0.15	*	0.61	*
	MEAS	−1.95	*	−3.47	*	−2.10	*	−2.99	*
MAG	SIM	0.00		1.93	*	1.76	*	1.66	*
	MEAS	−2.20	*	−2.08	*	−0.49	*	−1.94	*
MOD	SIM	−0.46	*	−0.22	*	−0.25	*	−0.64	*

The results of the Wilcoxon test are reported in Table 4. Significant differences were found between the two algorithms for both attitude and heading errors for the SIM scenario. On the other hand, for the MEAS scenario, the two algorithms provided significantly different results only for the attitude angle.

**Table 4:** Results (*Z* and *p*-values) of the comparison between Algorithms 1 and 2 for the SIM and MEAS scenarios. To improve the table readability/clarity, significant differences are also indicated with an asterisk.

Considered Scenario	Attitude			Heading		
	*Z*	*p*		*Z*	*p*	
SIM	−4.286	<0.001	*	−4.286	<0.001	*
MEAS	−2.143	0.032	*	−0.829	0.407	

## 4. Discussions

In the present study, a novel benchmarking method for SFAs performance assessment was presented and applied to a human movement analysis case study. In particular, the 3D pelvis orientation was estimated during a TUG test. A pool of hybrid scenarios, including both simulated and real MIMU data, was created and the combination of data associated to each scenario was fed to two different Kalman-based SFAs. The accuracy with which the pelvis orientation was estimated was then assessed by comparing the output of each algorithm with the reference output.

In Figure 4, the attitude curve exhibits two transitions at the beginning and the end of the test (about 3 s and 23 s), clearly corresponding to the sit-to-stand and stand-to-sit phases. The two 180° turns are also visible in the heading plot. Interestingly, both the attitude and heading error trends do not seem to be related with any of the activities mentioned above.

For both SFAs, the orientation errors were, on average, 3° and 4° for the attitude and heading, respectively (Figure 5). These errors can be considered rather large given the short test time duration (less than 20 s). In addition, it is clear that, for the task being shown, the GYR errors are highly correlated with the MEAS ones, suggesting a crucial role of the gyroscope errors in determining the performance of the SFA considered. To reduce orientation estimation errors, SFAs may be revised and tuned and, to do so, the information about the effect of the different noise sources characterizing each MIMU sensor is fundamental. The proposed benchmarking method aimed at providing a contribution in this respect. The following considerations are a concrete example of the information that can be derived by applying the proposed method for the assessment of a generic SFA.

For what concerns the intra-algorithm evaluation, a significant scenario effect was found for both Algorithms 1 and 2. This result was expected as the scenarios consisted of different combinations of simulated and real sensor data. The cascaded *post*-*hoc* analysis between the SIM and GYR scenarios revealed that using the simulated gyroscope data instead of the measured ones produced a significant effect for both attitude and heading accuracy, regardless of the considered SFA (Table 3). This means that the errors characterizing the gyroscope have a detrimental effect on both SFAs performance. In addition, both SFAs were found to take advantage of the simulated accelerometer and magnetometer data to partially correct the gyroscope-related errors (GYR *vs.* MEAS). This was always true except for the heading estimation obtained using Algorithm 1 (Table 3). Such a result may be explained by focusing on the inner structure of this specific algorithm, for which the angular velocity obtained from the gyroscope drives the accelerometer and magnetometer data pre-processing. Algorithm 1 is, thus, particularly sensitive to gyroscope imperfections and inconsistencies. Conversely, for what concerns Algorithm 2, accelerometer and magnetometer data are used to explicitly correct the quaternion predicted according to the gyroscope measurement. Therefore, the update step performed in this algorithm appears to be effective in correcting the prediction errors due to the gyroscope when the accelerometer and magnetometer are ideal. In any case, the GYR scenario is clearly the closest to the MEAS scenario, which represents the upper bound for the SFA error. This means that, both SFAs are more sensitive (both in terms of attitude and heading) to the gyroscope issues than to the accelerometer and magnetometer ones.

Significant differences were reported also between the ACC scenario and both the SIM and MEAS scenarios. On the one hand, the imperfections affecting the real accelerometer measurements degrade significantly the orientation estimates with respect to the SIM scenario. On the other hand, it has to be noted that both SFAs successfully exploit the other simulated sensors to reduce the errors due to these imperfections, as highlighted by the comparison between the ACC and the MEAS scenarios. Interestingly, significant differences between ACC and SIM were found also for the heading estimation, indicating that, although gravity measurements (from the accelerometer) do not convey heading information, errors in the accelerometer data cause errors in the heading estimation. This is also supported by the results of the comparison between the MOD and SIM scenarios: for both SFAs and both heading and attitude, a significant difference was observed between the two scenarios, which only differ for the presence of external accelerations (accelerometer inconsistency [3]). These two results are in accordance with the existing literature [24], where heading notoriously represents the most difficult degree of freedom to be estimated with MIMU data [6]. In fact, it is well known that attitude errors imply an additional error on heading [24]. Unfortunately, no algorithm design can prevent this effect.

When considering the heading estimation using both Algorithms 1 and 2, the errors associated to the MAG scenario were significantly different from those obtained for both the SIM and MEAS scenarios. On the other hand, for Algorithm 1, when the attitude is considered, no significant difference was observed between the MAG and SIM scenarios. This result may be explained as follows: within Algorithm 1 the actual orientation estimation is performed by the TRIAD block (see Figure 3), which uses the estimated gravity as the first (more reliable) direction. As a result, magnetic inconsistencies (due to ferromagnetic disturbances) are prevented from degrading significantly the attitude estimation. In other words, in Algorithm 1, the magnetometer readings are almost neglected for the attitude estimation. Conversely, for Algorithm 2, magnetic disturbances have a significant detrimental effect both on attitude and heading estimation, as magnetometer data are used for the attitude estimation as well. Given this result, it is recommendable to decouple the attitude estimation from the magnetometer measurements when designing SFA for orientation estimation targeted to indoor applications, where the hypothesis of uniform and constant magnetic field can be undermined.

With regard to the inter-algorithm comparison (Table 4), when considering the SIM scenario, a statistically different performance was exhibited by the two SFAs. In particular Algorithm 1 was characterized by smaller heading and attitude errors. This result can be explained by the different algorithm design and, in particular, by the fact that Algorithm 1 takes advantage of the linear approach to the Kalman filtering over the extended one implemented in Algorithm 2. On the other hand, this performance gap is much less evident when real sensor data are involved, as shown by the comparison of the two MEAS scenarios (Table 4). Even though a significant effect is reported for the attitude estimation in the MEAS scenarios, it should be more reasonably attributed to the exclusion of the magnetic measurements from the attitude estimation than to the linear estimation approach. In fact, no statistical differences were found for the heading estimation, for which the magnetometer is used by both methods. In other words, the different designs might indeed imply different estimation performances. However, these differences may be concealed by the inconsistencies and imperfections characterizing real data obtained from low grade MIMUs.

As a summary, the proposed benchmarking method has the benefit to allow for an improved understanding of the extent to which each sensor issue influences the final orientation estimate. This possibility is of paramount importance in the context of SFAs design to correctly consider the role of each sensor. As an example, by means of the proposed methodology the following considerations for the MIMU-based human motion tracking can be drawn: (1) the gyroscope errors appear to be the main error source for both the SFAs considered; (2) the processing of accelerometer data proposed in Algorithm 1 is promising because it reduces the detrimental effect of the external acceleration; (3) using the magnetometer data for the heading estimation only leads to more accurate attitude estimates. These considerations can then be used to improve the existing designs/tuning settings in accordance to the needs of the specific application.

## 5. Conclusions

In conclusion, the methodology presented in this paper allows to gain insight into the working principles of a generic sensor fusion algorithm. The proposed data hybridization process consists in combining real and ideal sensor data with the aim of addressing the main strengths and weaknesses of an SFA. Moreover, the results obtained with this methodology when applied to multiple SFAs allow highlighting their specific behavior with respect to different input data issues. The proposed benchmarking method was applied to a typical sensor fusion context in human movement analysis, namely 3D orientation estimation through MIMU data. The potential of the proposed methodology was thus exploited to reveal the main issues involved in this specific application context.

## Appendix A

In this appendix the two orientation estimators (Algorithms A1 and A2) evaluated and compared in this paper are detailed with a CLRS description, where:

- $x_{k}$ and ${\hat{x}}_{k}$ are, respectively, the real and estimated value of the state vector x;
- x^−k and x^+k are, respectively, the *a priori* and *a posteriori* estimate of the state vector x at the *k*-th time instant;
- $h^{n}$ and $h^{b}$ are the representation of the Earth’s magnetic field in the {n} and {b} reference frames, respectively;
- $g^{n}$ and $g^{b}$ are the representation of the Earth’s magnetic field in the {n} and {b} reference frames, respectively;
- σg, σa and σm are the standard deviations of the gyroscope, accelerometer and the magnetometer measurement, respectively;
- σb is a tuning parameter encoding the expected severity of the magnetic disturbances in method B;
- $c_{a}$ and $c_{b}$ are the Gauss-Markov parameters of the prediction models in method A;
- H, Q and R are, respectively, the extraction matrix, the process noise matrix and the measurement noise matrix in the Kalman filter;
- $T_{s}$ is the sampling time;
- × represents the cross product operator;
- ⊗ represents the quaternion product operator;
- $q^{bn}$ and $R^{bn}$ represents, respectively, the quaternion and the rotation matrix encoding the orientation from the {n} reference frame to the {b} reference frame.
- $I_{n}$ and $0_{n}$ are the n×n identity and null matrix;
- [p ×] is the cross product matrix for the vector $p={\left[\begin{array}{ccc} p_{1} & p_{2} & p_{3} \end{array}\right]}^{T}$, $[p\;\times ]=\left[\begin{array}{ccc} 0 & -p_{3} & p_{2}\\ p_{3} & 0 & -p_{1}\\ -p_{2} & p_{1} & 0 \end{array}\right]$;
- $\Omega (p)=\left[\begin{array}{cc} -\left[p\times \right] & p\\ -p^{T} & 0 \end{array}\right]$;
- Ξ(q)=[[qv×]+q4⋅I3qTv] with q=[q1q2q3q4]T=[qvq4]T being a generic unit quaternion;
- $\Psi \left(q,p\right)=\frac{\partial \left(q⊗\left[\begin{array}{c} p\\ 0 \end{array}\right]⊗q^{-1}\right)}{\partial \left(q\right)}$

**Algorithm A1**
**for** each measured sample from the sensors **do**
$\omega_{k}^{b}\leftarrow gyroscope\;sample$
***Prediction***
Predict gravity and acceleration (KF_acc_)
xkacc−=[g^kb−a^kb−]=[exp(−[ωk−1b×]⋅Ts) 0303ca⋅I3][g^k−1b+a^k−1b+]
Process noise covariance matrix (KF_acc_)
Qk−1acc=[[g^kb−×]0303cb⋅I3][I3⋅(σgTs)20303I3] [[g^kb−×]0303cb⋅I3]T
Predict Earth’s magnetic field and disturbances (KF_mag_)
xkmag−=[h^kb−d^kb−]=[exp(−[ωk−1b×]⋅Ts) 0303ca⋅I3][h^k−1b+d^k−1b+]
Process noise covariance matrix (KF_mag_)
Qk−1mag=[[h^kb−×]0303cb⋅I3][I3⋅(σgTs)20303I3] [[h^kb−×]0303cb⋅I3]T ***Update***:
H=Hmag=Hacc=[I3I3]
Measurement update (KF_acc_)
ykacc←accelerometer sample
Racc=I3⋅σ2a
x^kacc+← KalmanUpdate (xkacc−, Qk−1acc, ykacc, H, Racc)
Measurement update (KF_mag_)
ykmag←magnetometer sample
Rmag=I3⋅σ2m
x^kmag+← KalmanUpdate (xkacc−, Qk−1mag, ykmag, H, Rmag)
***Orientation computation (TRIAD algorithm)***
Normalize g^kb+ and h^kb+ to one
Mobs=[g^kb+g^kb+×h^kb+g^kb+×(g^kb+×h^kb+)]
$M_{ref}=\left[\begin{array}{ccc} g^{n} & g^{n}\times h^{n} & g^{n}\times \left(g^{n}\times h^{n}\right) \end{array}\right]$
${\hat{q}}_{k}^{bn}=matrixToQuaternion\left(M_{ref}M_{obs}^{T}\right)$
**end for**

**Algorithm A2**
**for** each measured sample from the sensors **do**
$\omega_{k}^{b}\leftarrow gyroscope\;sample$
***Prediction***
Predict the quaternion and the magnetic disturbance
x^k−=[q^kbn−b^knm−]=[exp(Ω(ωk−1b)Ts)0303I3][q^k−1bn+b^k−1nm+]
Process noise covariance matrix
Qk−1=[(σgTs2)2Ξ(q^k−1bn+)I3Ξ(q^k−1bn+)T04×303×4σ2b⋅I3]
***Update***:
compute R^bn−=quaternionToMatrix(q^bn−)
yk=[ykmagykacc],ykmag←magnetometer sampleykacc←accelerometer sample
Sensor data validation
Racc={I3⋅σ2a, if |yacc−R^bn−gn|<εacc∞, otherwise
Rmag={I3⋅σ2m, if |ymag−R^bn−(hn−b^knm−)|<εmag∞, otherwise
R=[RmagRacc]
Jacobian calculation
H=[Ψ(q^kbn−,hn+b^knm−)RbnΨ(q^kbn−,gn)03]
x^+←KalmanUpdate (x^−, $Q_{k-1}$, $y_{k}$, H, R)
**end for**

## References

[1] Corke P., Lobo J., Dias J. (2007). An Introduction to Inertial and Visual Sensing. Int. J. Robot. Res..

[2] Novak D., Riener R. (2015). A survey of sensor fusion methods in wearable robotics. Robot. Auton. Syst..

[3] Khaleghi B., Khamis A., Karray F.O., Razavi S.N. (2013). Multisensor data fusion: A review of the state-of-the-art. Inf. Fusion.

[4] Weiss S., Scaramuzza D., Siegwart R. (2011). Monocular-SLAM-based navigation for autonomous micro helicopters in GPS-denied environments. J. Field Robot..

[5] Lu Y., Michaels J.E. (2009). Feature extraction and sensor fusion for ultrasonic structural health monitoring under changing environmental conditions. IEEE Sens. J..

[6] Bergamini E., Ligorio G., Summa A., Vannozzi G., Cappozzo A., Sabatini A.M. (2014). Estimating Orientation Using Magnetic and Inertial Sensors and Different Sensor Fusion Approaches: Accuracy Assessment in Manual and Locomotion Tasks. Sensors.

[7] Mazomenos E., Biswas D., Cranny A., Rajan A., Maharatna K., Achner J., Klemke J., Jobges M., Ortmann S., Langendoerfer P. (2015). Detecting Elementary Arm Movements by Tracking Upper Limb Joint Angles with MARG Sensors. IEEE J. Biomed. Health Inform..

[8] Sabatini A.M. (2006). Inertial sensing in biomechanics: A survey of computational techniques bridging motion analysis and personal navigation. Computational Intelligence for Movement Sciences: Neural Networks and Other Emerging Techniques.

[9] Sabatini A.M. (2011). Estimating three-dimensional orientation of human body parts by inertial/magnetic sensing. Sensors.

[10] Ligorio G., Sabatini A.M. A Linear Kalman Filtering-Based Approach for 3D Orientation Estimation from Magnetic/Inertial Sensors. Proceedings of the IEEE International Conference on Multisensor Fusion and Information Integration.

[11] Harada T., Mori T., Sato T. (2007). Development of a tiny orientation estimation device to operate under motion and magnetic disturbance. Int. J. Robot. Res..

[12] Choukroun D., Bar-Itzhack I.Y., Oshman Y. (2006). Novel quaternion Kalman filter. IEEE Trans. Aerosp. Electron. Syst..

[13] Madgwick S.O., Harrison A.J., Vaidyanathan R. Estimation of IMU and MARG orientation using a gradient descent algorithm. Proceedings of the 2011 IEEE International Conference on Rehabilitation Robotics (ICORR).

[14] Mahony R., Hamel T., Pflimlin J.-M. (2008). Nonlinear Complementary Filters on the Special Orthogonal Group. IEEE Trans. Autom. Control.

[15] Titterton D., Weston J.L. (2004). Strapdown Inertial Navigation Technology.

[16] Bancroft J.B., Lachapelle G. Estimating MEMS gyroscope g-sensitivity errors in foot mounted navigation. Proceedings of the Ubiquitous Positioning, Indoor Navigation, and Location Based Service (UPINLBS).

[17] Strauss O. Filtering and fusing compass and gyrometer data using Guess filter. Proceedings of the Sixth IEEE International Conference on Fuzzy Systems.

[18] Winter D.A. (2009). Biomechanics and Motor Control of Human Movement.

[19] Chardonnens J., Favre J., Aminian K. (2012). An effortless procedure to align the local frame of an inertial measurement unit to the local frame of another motion capture system. J. Biomech..

[20] Ligorio G., Sabatini A.M. (2015). A Simulation Environment for Benchmarking Sensor Fusion-Based Pose Estimators. Sensors.

[21] Shuster M.D. (1993). A survey of attitude representations. Navigation.

[22] Black H.D. (1964). A passive system for determining the attitude of a satellite. AIAA J..

[23] Faber G.S., Chang C.-C., Rizun P., Dennerlein J.T. (2013). A novel method for assessing the 3-D orientation accuracy of inertial/magnetic sensors. J. Biomech..

[24] Caruso M.J. Applications of magnetic sensors for low cost compass systems. Proceedings of the Position Location and Navigation Symposium.

